# Influence of sex hormone use on sleep architecture in a transgender cohort

**DOI:** 10.1093/sleep/zsad249

**Published:** 2023-09-16

**Authors:** Margot W L Morssinkhof, Ysbrand D van der Werf, Odile A van den Heuvel, Daan A van den Ende, Karin van der Tuuk, Martin den Heijer, Birit F P Broekman

**Affiliations:** Department of Psychiatry, Amsterdam UMC, Location Vrije Universiteit Amsterdam, The Netherlands; Department of Endocrinology and Metabolism, Amsterdam UMC, Location Vrije Universiteit Amsterdam, The Netherlands; Department of Psychiatry and Medical Psychology, OLVG, Amsterdam, The Netherlands; Center of Expertise on Gender Dysphoria, Amsterdam UMC, Vrije Universiteit Amsterdam, The Netherlands; Department of Anatomy and Neurosciences, Amsterdam UMC, Vrije Universiteit Amsterdam, Amsterdam, The Netherlands; Amsterdam Neuroscience, Compulsivity Impulsivity and Attention, Amsterdam, The Netherlands; Department of Psychiatry, Amsterdam UMC, Location Vrije Universiteit Amsterdam, The Netherlands; Department of Anatomy and Neurosciences, Amsterdam UMC, Vrije Universiteit Amsterdam, Amsterdam, The Netherlands; Amsterdam Neuroscience, Compulsivity Impulsivity and Attention, Amsterdam, The Netherlands; Remote Patient Monitoring & Chronic Care, Philips, Eindhoven, The Netherlands; Department of Obstetrics and Gynecology, University Medical Centre Groningen, Groningen, The Netherlands; Department of Endocrinology and Metabolism, Amsterdam UMC, Location Vrije Universiteit Amsterdam, The Netherlands; Center of Expertise on Gender Dysphoria, Amsterdam UMC, Vrije Universiteit Amsterdam, The Netherlands; Department of Psychiatry, Amsterdam UMC, Location Vrije Universiteit Amsterdam, The Netherlands; Department of Psychiatry and Medical Psychology, OLVG, Amsterdam, The Netherlands; Amsterdam Public Health, Mental Health Program, Amsterdam, The Netherlands

**Keywords:** estradiol, testosterone, transgender, sleep EEG

## Abstract

**Study Objectives:**

Sex differences in sleep architecture are well-documented, with females experiencing longer total sleep time, more slow wave sleep (SWS), and shorter Rapid Eye Movement (REM) sleep duration than males. Although studies imply that sex hormones could affect sleep, research on exogenous sex hormones on sleep architecture is still inconclusive. This study examined sleep architecture changes in transgender individuals after 3 months of gender-affirming hormone therapy (GAHT).

**Methods:**

We assessed sleep architecture in 73 transgender individuals: 38 transmasculine participants who started using testosterone and 35 transfeminine participants who started using estrogens and antiandrogens. Sleep architecture was measured before GAHT and after 3 months of GAHT for 7 nights using an ambulatory single-electrode sleep EEG device. Changes in sleep architecture were analyzed using linear mixed models, and non-normally distributed outcomes were log-transformed and reported as percentages.

**Results:**

In transmasculine participants, SWS decreased by 7 minutes (95% CI: −12; −3) and 1.7% (95% CI: −3%; −0.5%), REM sleep latency decreased by 39% (95% CI: −52%; −22%) and REM sleep duration increased by 17 minutes (95% CI: 7; 26) after 3 months of GAHT. In transfeminine participants, sleep architecture showed no significant changes after 3 months of GAHT.

**Conclusions:**

Sleep architecture changes after 3 months of masculinizing GAHT in line with sleep in cisgender males, while it shows no changes after feminizing GAHT. The sex-specific nature of these changes raises new questions about sex hormones and sleep. Future research should focus on studying possible underlying neural mechanisms and clinical consequences of these changes.

## Graphical Abstract

**Figure:**
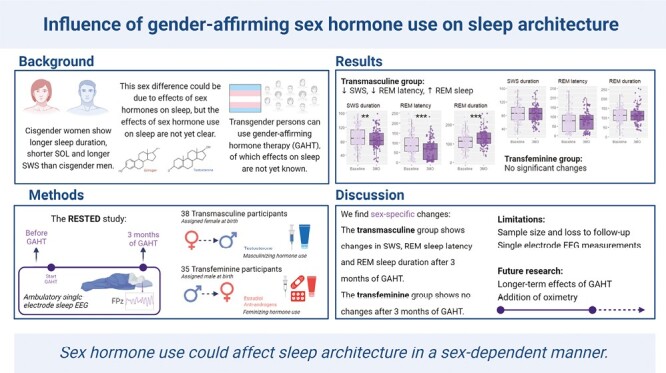

**Statement of Significance**

Sleep architecture shows differences between men and women, with women showing longer sleep, longer slow wave sleep, and shorter rapid eye movement sleep than men. Although studies in rodents and patients with hormonal conditions indicate that sex hormones can alter sleep architecture, the effects of sex hormone use on sleep architecture in healthy humans are not entirely clear. This study examined effects of 3 months of gender-affirming hormone use in transgender individuals. Results show that testosterone use in persons assigned female at birth resulted in sleep architecture changes similar to cisgender males, whereas estradiol- and antiandrogen use by persons assigned male at birth did not change sleep architecture. These novel findings indicate that sex hormones could change sleep architecture in a sex-specific manner, warranting further studies into causal mechanisms underlying these changes.

## Introduction

There are many known physiological differences between men and women, but one of the lesser-known sex differences is found in sleep architecture. Sleep architecture, which can be measured with sleep electroencephalography (EEG), refers to the organization of sleep into sleep depth and sleep stages. When measuring sleep with EEG, healthy women generally show better sleep quality than healthy men (e.g. longer total sleep time (TST), less time awake during the night, longer deep sleep, and longer Rapid Eye Movement (REM) latency [1, 2]). Although researchers have intensively studied sleep architecture and the underlying neural mechanisms, the cause of these sex differences in sleep architecture is still not fully understood.

One of the hypothesized mechanisms underlying the sex differences in sleep architecture is attributed to the effect of sex hormones. In mice, sex differences in sleep architecture are eliminated after gonadectomy, which results in the loss of sex steroid production [3]. Female mice also show longer REM sleep durations after gonadectomy, and restoration of estradiol levels through supplementation also restores the shorter REM sleep duration [4, 5]. There are indications that the influence of sex steroids is sex-specific: an experiment using administration of estrogen and testosterone in female and male rats shows that female, but not male, rats show changes in sleep (e.g. increased wakefulness, changes in REM sleep) after administration of either hormone [4].

In humans, knowledge of exogenous sex hormones and sleep architecture in participants without hormonal disorders is still scarce. Studies have mainly focused on clinical groups using hormone therapy, such as women during perimenopause and hypogonadal men. A few studies in healthy reproductive-age persons showed indications of sex hormone effects on sleep architecture. In healthy men with induced hypogonadism, testosterone supplementation resulted in more deep sleep and a longer REM sleep latency compared to placebo [6]. Similarly, estrogen therapy in hypogonadal females resulted in decreased sleep onset latency (SOL) and longer REM sleep duration compared to placebo [7]. Findings on progesterone are less consistent: progesterone administration was found to have a sedative effect in healthy males and females [8] but also reduced slow wave sleep (SWS) duration in males [9]. Administration of a progesterone antagonist in healthy males resulted in increased SOL and more time spent awake during the night [10].

One of the limitations in the field of sex hormone use is that most studies that examine have mainly focused on effects of short-term hormone administration, ranging from days to weeks of hormone use, and hormone use within the same sex (e.g. use of estrogens and progestins in females, or testosterone in males). Furthermore, it is difficult to examine the causal role of sex hormone use in groups with sex hormone conditions, including peri- and postmenopausal females, hypogonadal males, and females with polycystic ovary syndrome. These conditions are already known to affect sleep, and therapy in the form of sex hormone use could restore healthy hormone levels and therefore improve sleep [11–13]. This makes it difficult to disentangle whether the observed sex differences in sleep architecture in the population are driven by sex hormones, or by other biological sex-specific factors like sex chromosomes or developmental sex differences. One way of gaining insight into the effects of sex hormones on human sleep would be to study the effects of male sex hormones (e.g. testosterone) in females and the effects of female sex hormones (e.g. estrogen) in males, but this is difficult to do in an experimental setting.

Transgender users of gender-affirming hormone therapy (GAHT) use sex hormones to masculinize or feminize their bodies, to better align their physical state with their gender identity [14]. The study of changes in sleep architecture after GAHT use can offer insight into the direct effect of sex hormone use on sleep architecture. The use of GAHT in transfeminine persons assigned male at birth most often consists of antiandrogens and estrogens which act to feminize the body, and the use of GAHT in transmasculine persons assigned female at birth (AFAB) consists of testosterone use which acts to masculinize the body. Since GAHT use commonly results in sex steroid levels comparable to the opposite sex, this enables us to specifically examine the effects of healthy physiological levels of sex hormones on sleep.

Thus far, the effect of GAHT on sleep architecture is unclear. Only one study examined effects of feminizing GAHT in seven transgender women [15], and they found that participants showed more light sleep after 3 months of estradiol and cyproterone acetate. This study, however, was limited both in sample size and in participant selection, since they only included transgender women.

Therefore, this study aims to examine the changes in sleep architecture after GAHT use. We examine whether characteristics of sleep architecture (e.g. TST, wakefulness after sleep onset, SWS duration and frequency, number of arousals, number of awakenings, REM sleep duration, and REM sleep latency) change after three of either feminizing or masculinizing hormone use. We hypothesize that testosterone use in transmasculine persons is associated with shorter total sleep duration, more wakefulness during the night and more arousals and awakenings, shorter SWS duration, and shorter REM sleep latency, and that estrogen and antiandrogen use by transfeminine persons is associated with longer total sleep duration, less wakefulness during the night and less arousals and awakenings, longer SWS duration and longer REM sleep latency.

## Methods

### Study setting

Data for this study was obtained within the RESTED (Relationship between Emotions and Sleep in Transgender Persons: Endocrinology and Depression) study, which is an observational prospective study aimed at studying changes in mood and sleep in transgender hormone users during the first year of GAHT. The RESTED study was conducted within the transgender healthcare clinics of the Amsterdam University Medical Center (Amsterdam UMC) and the University Medical Center Groningen and included participants from January 2020 until September 2022. The RESTED study received a declaration stating that Medical Research Involving Human Subjects Act (WMO) did not apply to this data collection (study id: 2019.353). The study was performed in accordance with good clinical practice guidelines and the World Medical Association Code of Ethics (Declaration of Helsinki). All participants provided informed consent for study participation and for use of their medical information for research purposes.

### Participants and measurements

All participants were approached and included before the start of GAHT. Participants were eligible to participate in the RESTED study if they were aged between 18 and 50 years, if they were diagnosed with gender dysphoria, and if they had never used gender-affirming hormones before. They were not included if they had a diagnosed sleep disorder (e.g. obstructive sleep apnea, clinical insomnia, or parasomnias), or if they used benzodiazepines or barbiturates at time of the study.

### Gender-affirming hormone therapy

All participants started using GAHT after the baseline measurement of the study, and participants were treated with GAHT according to the WPATH guidelines [16]. Transmasculine participants, who were assigned female at birth (AFAB), all started using masculinizing hormones in the form of testosterone. These hormones were either administered via testosterone gel (daily dose of 40.5 mg), testosterone esters (250 mg every 3 weeks), or testosterone undecanoate (1000 mg every 12 weeks). A number of transmasculine participants also used progestins (lynestrenol, 5 mg; levonorgestrel IUD, 52mg; medroxyprogesterone, 150 mg) or combined hormonal contraceptives, consisting of progestins and estrogens (ethinylestradiol/levonorgestrel, 0.03/0.15 mg; ethinylestradiol/drospirenon 0.02/3 mg) to suppress their menstruation.

Transfeminine participants, who were assigned male at birth, started using feminizing hormones, consisting of antiandrogens, either in the form of oral cyproterone acetate (CPA; daily dose of 10 mg) or intramuscular injections of gonadotropin-releasing hormone analogs (GnRH analogs), either short-working (triptorelin 3.75 mg per 4 weeks, or leuproreline, 3.75 mg per 4 weeks) or long-working (triptorelin 11.25 mg per 12 weeks), combined with estradiol, which was either administered orally (estradiol valerate, 2 mg twice daily) or transdermally through estradiol gel (0.06% 1.5 mg daily) or estradiol patches (100 mcg per 24 hours, twice a week). For the current study, transfeminine participants were excluded if they only started using estrogens (*n* = 1) or antiandrogens (*n* = 1) but not both at the start of GAHT.

### Demographic and clinical characteristics

To collect information on participants’ demographic characteristics, data from electronic patient files was combined with data from study surveys. At each outpatient clinic appointment participants were asked about medication use and intoxications (e.g. smoking, alcohol, and drug use). As part of regular care, presence of possible psychiatric diagnoses was tested using the MINI+ (Mini International Neuropsychiatric Interview [17]), a structured clinical interview, at the intake appointment of the clinic. In both participating centers, serum hormone levels were measured as part of regular care at the start of GAHT use, after 3 months of GAHT use, and after 12 months of GAHT use. In both the Amsterdam UMC and the University Medical Center Groningen, serum testosterone measurements were conducted using liquid chromatography-tandem mass spectrometry (LC-MS/MS) with a lower limit of quantitation of 0.1 nmol/L, and an inter-assay coefficient of variation of 4% to 9%. Serum estradiol measurements in both centers were conducted using LC-MS/MS with a lower limit of quantitation of 20 pmol/L and an inter-assay coefficient of variation of <7%.

### Sleep measurements

The study consisted of three periods of sleep measurements of 1 week each: the first measurement was conducted before the start of GAHT, the second measurement after 3 months of GAHT, and the third measurement after 12 months of GAHT. In each measurement week, participants were asked to record sleep architecture for seven nights using an ambulatory single-electrode EEG sleep measurement device (Smartsleep, Philips, the Netherlands). Self-reported sleep was measured with the consensus sleep diary [18] and self-reporting questionnaires on sleep (the Pittsburgh Sleep Quality Index [PSQI][19], and Insomnia Severity Index [ISI] [20]).

### Sleep diaries and sleep questionnaires

The consensus sleep diary [18] (CSD) is a standardized diary to track information on daily sleep patterns, including sleep-specific questions about time spent in bed, TST, SOL, and amount of times and duration of wakefulness during the night. The CSD also asks about factors affecting sleep, such as coffee consumption, alcohol consumption, and use of substances or medication that affect sleep. For the current study, questions were added to the CSD which inquired about experiences using the sleep tracker. A comment field was added in which participants could note comments about the measurement night, including remarks on the sleep tracker.

To assess sleep quality and symptoms of insomnia, the PSQI and ISI were used at every measurement. The PSQI is a 19-item questionnaire that broadly assesses components of sleep quality, including sleep duration, sleep quality, sleep disturbances, and daily impact of sleep problems [19]. It is scored into seven subscores, which are then added up to form a sum score (range 0 to 21). The PSQI has an established cutoff score of five, with a score of 5 or lower indicating “good” sleep and a score higher than five indicating “poor” sleep. The ISI is a 7-item questionnaire that inquires about symptoms of insomnia in the previous 2 weeks [21]. The seven items are all scored from 0 to 4, resulting in a sum score from 0 to 28. There are clinical cutoffs for the ISI scores, with a score below seven indicating no presence of insomnia, 8 to 14 indicating subclinical insomnia, 15 to 21 indicating clinical insomnia, and a score over 21 indicating severe insomnia. The items in the ISI correspond to the DSM-5 defined criteria for clinical insomnia, and are a widely used tool to assess the impact of insomnia symptoms. To assess perceived stress and depressive symptoms, the Perceived Stress Scale [22] (PSS) and Inventory of Depressive Symptomatology-Self Report [23] (IDS-SR) were used. Questionnaire results for both were used to assess the presence of selection bias in the sleep EEG measurements as displayed in Supplementary Table S2.

### Sleep architecture

Sleep architecture, consisting of SOL, TST, wake after sleep onset (WASO), sleep efficiency (SE), SWS duration, number of arousals (NRA), number of interruptions (NRI; >5 minutes awake), REM sleep duration and REM sleep latency was measured using the EEG sleep measurement device with an embedded automated sleep stager. This sleep measurement device uses dry single-lead EEG for measuring sleep, with electrodes at Fpz and M1. Based on this single-channel EEG signal, the embedded stager can differentiate wakefulness, light sleep (i.e. phase 1 and 2), deep sleep (i.e. phase three sleep or SWS), and REM sleep, with excellent performance in validation studies compared to manual scoring: κ = 0.65–0.67 on the same single channel from the device (close to the inter scorer agreement, κ = 0.69) [24], and even better agreement for the automatic stager compared to manual scoring from EEG and EOG signals, and from full PSG (κ = 0.73 and κ = 0.76, respectively) [25]. Participants were asked to record their sleep at home. After completing a week of measurements, the device was brought or sent back to the hospital and the data on the device was uploaded to the study database by the local research team.

Measurements were cleaned to exclude incomplete nights or nights with poor measurement quality: a measurement night was considered incomplete if the participant reported that they took off the headband during the night in the sleep diary, if the measured TST, WASO, and SOL together were shorter than 240 minutes or if the contact impedance was higher than the devices’ internal threshold limit threshold for good quality recordings. For the analyses, the first night of each sleep architecture measurement week was not used for analysis, since the first night was considered a habituation night. The SE was calculated by dividing the TST by the total duration of the sleep episode (e.g. the sum of the TST, SOL, and WASO) and multiplying this ratio by 100 to get a percentage.

In 26 of the 121 measurements, the device was inadvertently set in active mode (as described in [24]). The active mode is designed to increase slow wave amplitude in sleep-restricted populations, but was shown to not affect sleep architecture (e.g. total sleep duration, WASO, SWS) [24]. Sensitivity analyses incorporating device setting as covariate show that the active mode did not significantly affect measurements, as shown in Supplementary Materials 2. Hence, upon consideration, we deemed this not to be a significant factor for our research questions.

### Statistical analyses

Continuous outcomes were summarized with means and standard deviations if they were normally distributed and medians, 25th percentile, and 75th percentile if they were not normally distributed. Categorical outcomes were summarized based on counts and percentages. Outcome variables with a non-normal distribution in residuals were log-transformed to ensure that the data met the assumptions of the statistical analyses.

Changes in sleep architecture were analyzed using linear mixed models in Rstudio (version 1.3.1093) using packages “lme4” [26] and lmerTest [27]. To estimate changes in sleep architecture after 3 months of GAHT, a model was constructed with the sleep outcomes per night (SOL, TST, WASO, SE, NRI, NRA, SWS, SWS%, and REM sleep latency and REM sleep duration) as outcome variables, timepoint (e.g. baseline or 3 months of GAHT) as the fixed predictor and a random intercept per participant to adjust for repeated measures within participants. Since a number of participants reported the use of psychotropic medication, we conducted an additional sensitivity analysis in which we adjusted for the use of psychotropic medication by adding this as a covariate in the model. To correct for repeated testing of the main results, Bonferroni correction was applied to all the main analyses, resulting in a corrected *P*-value threshold of 0.0025.

## Results

### Demographic and clinical characteristics

**Figure 1.**
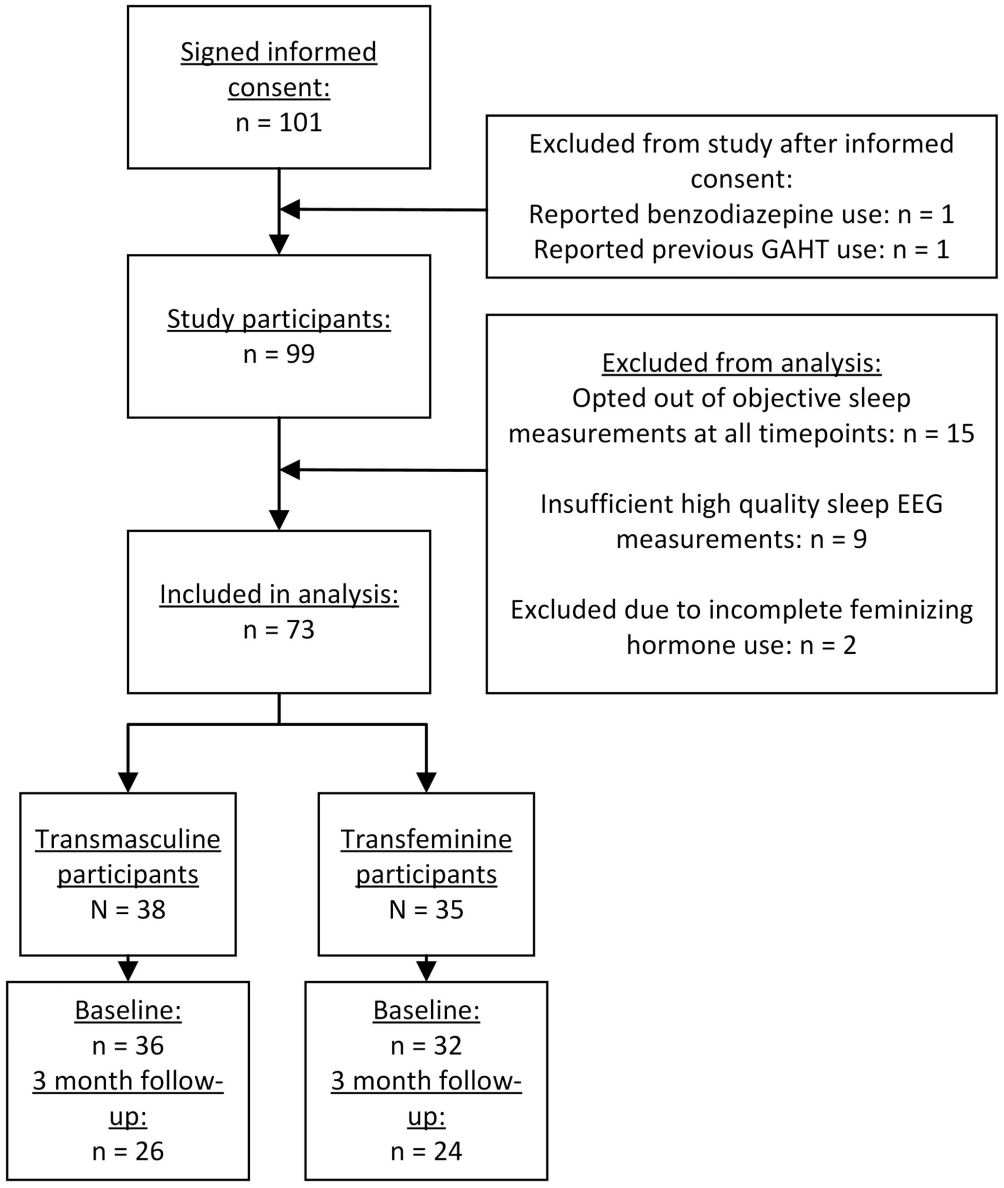
Inclusion flowchart of the RESTED study, showing inclusion rates and sample sizes per group and timepoint in the study.

Figure 1 displays the sample size of the full RESTED study and the sample size of the current study. The total study sample of the current study consists of 38 transmasculine participants, of whom 36 contributed data in the baseline measurement and 26 contributed data at the 3-month follow-up, and 35 transfeminine participants, of whom 32 contributed data to the baseline measurement and 24 contributed data to the 3-month follow-up measurement. In order to use the available data in the most optimal manner, all available data were used, including data from participants whose data were only present in one of the two measurements. As displayed in Table 1, transmasculine participants’ median age at baseline was 23, and transfeminine participants’ median age at baseline was 26. At baseline, 17% of transmasculine and 12% of transfeminine participants used psychotropic medication, most commonly antidepressants (11% in transmasculine and 9% in transfeminine participants) and stimulants (6% in transmasculine and 6% in transfeminine participants).

**Table 1.** Demographic and Clinical Characteristics at the Baseline Measurement and 3-month Follow-up

Measurement		Transmasculine participants (*n* = 38)		Transfeminine participants (*n* = 35)	
		Baseline *n* = 36	3 months *n* = 26	Baseline *n* = 32	3 months *n* = 24
Age	*median, 25th and 75th percentile*	23 (21 to 25)	22 (20 to 24.8)	26 (23.8 to 29.3)	27 (24 to 33)
Center (*n*, %)	*Amsterdam UMC*	31 (86.1%)	23 (88.4%)	29 (90.6%)	20 (83.3%)
	*UMCG*	5 (13.9%)	3 (11.5%)	3 (9.4%)	4 (16.7%)
Psychotropic medication use (*n*, %)	*Any psychotropic medication*	6 (16.7%)	5 (19.2%)	4 (12.5%)	4 (16.7%)
	*Antidepressants*	4 (11.1%)	3 (11.5%)	3 (9.4%)	1 (4.2%)
	*Antipsychotics*	1 (2.8%)	0 (0%)	0 (0%)	0 (0%)
	*Anxiolytics*	0 (0%)	0 (0%)	0 (0%)	0 (0%)
	*Stimulants*	2 (5.6%)	2 (8.3%)	2 (6.3%)	2 (8.3%)
	*Mood stabilizers*	0 (0%)	0 (0%)	0 (0%)	0 (0%)
Testosterone serum level (nmol/L; median, IQR) ^1^	*No cycle regulation use (in TM participants)*	0.9 (0.8 to 1.2)	16 (9 to 23)	14 (10 to 17)	0.6 (0.4 to 0.8)
	*Cycle regulation use (in TM participants)*	0.6 (0.4 to 0.9)	15 (12 to 18)	—	—
Estradiol serum level (pmol/L, median; IQR) ^1^	*No cycle regulation use (in TM participants)*	193 (135 to 291)	153 (109 to 202)	76 (63 to 90)	280 (177 to 378)
	*Cycle regulation use (in TM participants)*	77 (46 to 104)	99 (71 to 166)	—	—
PSQI score	*Median, 25th and 75th percentile*	7 (3.5 to 9.5)	5 (4 to 7) [2]	6 (4 to 7)	5 (3.5 to 7)
	*Mean, SD*	6.58 (3.22)	5.52 (2.0)	5.90 (2.62)	5.17 (2.29)
ISI score	*M* *edian, 25th and 75th percentile*	6 (4.5 to 10)	6 (4 to 7.5) [2]	5.5 (3 to 9)	5.5 (3 to 10.3)
	*Mean, SD*	7.54 (4.85)	5.96 (3.43)	6.31 (4.65)	6.96 (5.05)

^1^Reference ranges in cisgender persons: testosterone 9 nmol/L to 30 nmol/L and estradiol 12 pmol/L to 126 pmol/L in cisgender males and testosterone 0.3 to 1.6 nmol/L and 31 pmol/L to 2864 pmol/L in cisgender females (Amsterdam UMC Endocrinologisch Lab, z.d.; Verdonk et al., 2019).

According to WPATH SOC 8 guidelines, treatment providers should aim for serum levels associated with users’ gender identity, e.g. transmasculine GAHT users meeting the cis male reference range and transfeminine GAHT users meeting the cis female reference range.

^2^Significant change in the PSQI and ISI compared to baseline in the TM group. The PSQI is estimated to reduce by 0.89 points (95% CI: −1.62 to 0.18; *p* = 0.022) and the ISI is estimated to reduce by 1.37 points (95% CI: −2.38 to −0.39, *p* = 0.012).

At baseline, 50% of transmasculine participants used a form of cycle regulation: 11.1% used progestin-only oral forms, 22.2% used progestin-only non-oral forms, and 16.7% used a combined oral contraceptive with estradiol and progestins. After starting GAHT use, 89% of the transmasculine participants used testosterone in the form of testosterone gel, and 11% used testosterone in the form of short-acting testosterone ester injections. In the transfeminine participants after GAHT start, 53% used estradiol in the form of oral estradiol tablets, 38% used transdermal estradiol patches and 9% used transdermal estradiol gel. All transfeminine participants used combined estradiol use with anti-androgen use, with 84% of transfeminine participants using short-acting triptorelin injections, 9.4% using short-acting leuproreline injections and 9.4% using oral cyproterone acetate. All GAHT forms at each follow-up are also reported in Supplementary Table S1.

### Changes in sleep architecture after GAHT

#### Transmasculine participants.

**Figure 2.**
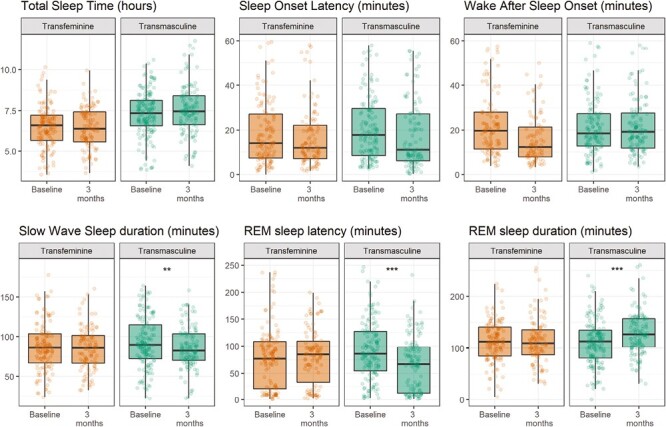
Box plots displaying medians and interquartile ranges (IQRs) of the outcome variables. Dots display the spread of the individual measurement nights; a number of outliers outside of the IQR are not displayed. ** = *p* < 0.005, *** = *p* < 0.0005.

A total of 38 transmasculine participants were included in the data of the current study: 36 participants contributed 146 measurement nights to the baseline measurement, and 26 participants contributed 118 measurement nights to the 3-month follow-up measurement. As shown in Table 2, statistical analyses show that after 3 months of GAHT, results show a trend towards a decreased SOL (−20.5%, *p* = 0.051), no significant change in the TST and a trend towards a decrease in WASO (−14.2%, *p* = 0.085). After 3 months of GAHT, the number of interruptions (NRI) showed no significant change, but the number of arousals (NRA) decreased (−10.8%, *p* = 0.044) and the SE improved (2.3%, *p* = 0.036). Furthermore, after 3 months of GAHT SWS duration (−7.4 minutes, *p* = 0.003) and SWS percentage (−1.7%, *p* = 0.007) decreased, REM sleep latency decreased (−38.6%, *p* = 0.0001) and REM sleep duration increased (16.5 minutes, *p* = 0.0008). Correction for multiple testing showed that the changes in REM sleep latency and REM sleep duration remained significant after correction for repeated testing (*p* = 0.0025). See Figure 2 for visualization of the sleep architecture outcomes.

**Table 2.** Sleep Architecture Before GAHT Use and After 3 Months of GAHT Use

	Transmasculine participants				Transfeminine participants			
Timepoint	Baseline	3 months	Statistical difference^1^		Baseline	3 months	Statistical difference ^1^	
*n* (mean number of valid nights per participant)	36 (4.3)	26 (4.5)	Unadjusted model	Adjusted model ^2^	32 (3.9)	24 (4.1)	Unadjusted model	Adjusted model^2^
SOL (minutes)	19.5 (9.7 to 34.9)	13.4 (7.0 to 34.6)	−20.5% (37.9% to 0.0%) *p* = 0.051	−24.2% (−10.3% to −3.9%) *p* = 0.023	14.97 (7.5 to 30.7)	13.1 (7.4 to 24.4)	−1.12% (−5.93% to 3.86%) *p* = 0.65	−1.11% (−5.9% to 3.8%) *p* = 0.66
TST (hours)	7.3 (1.41)	7.53 (1.52)	0.17 (−0.15 to 0.49) *p* = 0.29	0.20 (−0.12 to 0.53) *p* = 0.23	6.52 (1.32)	6.47 (1.28)	0.006 (−0.31 to 0.32) *p* = 0.97	0.005 (−0.30 to 0.32) *p* = 0.97
WASO (minutes)	21.9 (14.0 to 38.7)	19.6 (12.6 to 29.7)	−14.2% (−27.9% to 2.1%) *p* = 0.085	−14.6% (−28.7% to 2.4%) *p* = 0.087	21.06 (11.7 to 39.7)	14.98 (8.29 to 31.60)	−8.64% (−24.0% to 9.55%) *p* = 0.33	−8.6% (−24.0% to 9.52%) *p* = 0.34
SE (%)	89.9 (85.6 to 94.5)	91.9 (85.6 to 94.5)	2.3% (0.1% to 4.6%) *p* = 0.044	2.5% (0.2% to 4.9%) *p* = 0.036	90.4 (83.6 to 93.6)	92.4 (86.4 to 95.2)	0.9% (−2.3% to 2.5%) *p* = 0.94	0.1% (−2.3% to 2.5%) *p* = 0.95
NRI (*n*)	1 (0 to 2)	1 (0 to 2)	−7.7% (−18.% to 4.9%) *p* = 0.22	−8.0% (−19.4% to 5.0%) *p* = 0.22	1 (0 to 2)	0.5 (0 to 1)	−3.23% (−16.1% to 11.5%) *p* = 0.65	−3.13% (−16.1% to 11.5%) *p* = 0.66
NRA (*n*)	29 (21 to 44)	25 (19 to 35.5)	−10.8% (−20.2% to −0.4%) *p* = 0.044	−10.4% (−20.0% to 0.4%) *p* = 0.059	28.5 (18 to 41.25)	24 (17 to 40)	−0.96% (−12.6% to 12.3%) *p* = 0.88	−0.96% (−12.7% to 12.3%) *p* = 0.88
SWS (minutes)	90.6 (34.2)	85.2 (27)	−7.4 (−12.2 to −2.6) *p* = 0.0028	−8.2 (−13.1 to −3.2) *p* = 0.001 ^3^	87 (30.6)	84.6 (28.2)	−3.41 (−8.47 to 1.66) *p* = 0.19	−3.40 (−8.5 to 1.7) *p* = 0.19
% SWS (relative to TST)	21.0 (7.8)	19.5 (7.0)	−1.7 (−3.0 to −0.5) *p* = 0.0073	−2.1 (−3.4 to −0.8) *p* = 0.0017^3^	22.75 (8.36)	22.2 (7.58)	−1.00 (−2.70 to 0.7) *p* = 0.25	−0.99 (−2.7 to 0.7) *p* = 0.25
REM sleep latency (minutes)	87 (58 to 129)	68 (12.0 to 99.5)	−38.6% (−52.1% to −21.6%) *p* = 0.0001^3^	−41.1% (54.4% to −24.1%) *p* = 0.00006^3^	78 (21.0 to 109.5)	85 (33 to 109)	21.51% (−8.60% to 60.8%) *p* = 0.175	21.6% (−8.70% to 60.6%) *p* = 0.174
REM sleep duration (minutes)	111.3 (44.2)	128.5 (41.6)	16.5 (6.9 to 26.0) *p* = 0.00085^3^	18.2 (8.3 to 28.0) *p* = 0.0004^3^.	113.92 (42.1)	113.88 (44.4)	−2.16 (−11.92 to 7.63) *p* = 0.66	−2.21 (−11.9 to 7.57) *p* = 0.66

Outcomes at baseline and 3 months are reported in mean and *SD* if normally distributed and median, 25th and 75th percentile if not normally distributed. SOL, sleep onset latency; TST, total sleep time; WASO, wake after sleep onset; SE, sleep efficiency; NRI, number of interruptions; NRA, number of arousals; SWS, slow wave sleep; REM, rapid eye movement.

^1^Estimate, 95% confidence interval, and *P*-value are reported. Estimates for variables that were non-normally distributed (e.g. SOL, WASO, SE, NRI, and NRA) were log-transformed and are reported in % of change.

^2^Adjusted for the use of psychotropic medication.

^3^Significant after Bonferroni correction for repeated testing (*p* = 0.0025).

#### Transfeminine participants.

A total of 35 transfeminine participants were included in the data of the current study: 32 participants contributed 124 measurement nights to the baseline measurement, and 24 participants contributed 102 nights of measurements to the 3-month follow-up measurement. As shown in Table 2, statistical analyses show no significant changes after 3 months of GAHT compared to baseline (Figure 2).

#### Psychotropic medication.

The incorporation of psychotropic medication use as an additional covariate did not significantly affect the direction or magnitude of the results, as displayed in Table 2 in the adjusted model results.

## Discussion

This study was the first to prospectively study effects of both masculinizing and feminizing sex hormones on sleep architecture in transgender persons. We found that 3 months of masculine sex hormone use resulted in decreased SWS duration, decreased REM sleep latency, and increased REM sleep duration. However, 3 months of feminine sex hormone use showed no significant effects on sleep architecture. The results support our hypothesis that masculinizing hormones in persons AFAB change the sleep architecture towards the opposite sex. However, feminizing hormones in persons assigned to males at birth does not change sleep architecture, which does not support our hypothesis.

In the transmasculine participants, the changes in REM sleep latency and duration and in SWS duration are notable. REM sleep latency shifts from a median of 87 to 68 minutes and the REM sleep duration shows an increase from a median of 111 minutes to 128 minutes. Considering a REM sleep duration of 21% to 30% in proportion of total sleep is considered healthy [28], these strong changes could be clinically relevant. The changes in SWS duration are small and not consistently significant after correction for repeated testing, with a 7-minute decrease in the raw SWS duration (corresponding to a Cohen’s d of 0.16) or 1.7% decrease in SWS percentage (corresponding to a Cohen’s d of 0.23), but very consistent across participants. This shows that the use of testosterone in persons AFAB could affect SWS duration. Considering that the typically healthy range of SWS is from 16% to 20% [28], and participants showed a decrease from 21% to 19.5%, we would deem this change to be of interest for sleep science but of limited clinical meaning.

Our results in the transfeminine participants mostly are in line with previous work conducted by [15]. This study assessed the effects of 3 months of feminizing GAHT, specifically estrogen and cyproterone acetate, in seven transgender women. They found no significant changes in most sleep stages, but reported a specific increase in light (N1) sleep, from 33 minutes to 51 minutes. Our current study did not assess light sleep duration, and we could therefore not replicate the finding in N1 sleep. However, this study by Künzel and colleagues found no other changes in sleep architecture, which is in line with our current findings.

It is important to note that, although transmasculine participants were using testosterone, it is not clear whether the androgenic effects of testosterone are also causing the changes in sleep. Testosterone can affect androgenic pathways, as testosterone or via conversion into dihydrotestosterone (DHT), or it is aromatized locally into estradiol and affects estrogen receptors [29]. Previous work showed that in female rats, treatment with testosterone affected sleep, but treatment with DHT, which cannot be aromatized into estradiol, did not [4]. Therefore, it is also possible that in our participants, the reported effects are not exclusively caused by the androgenic effects of testosterone, but that the administered testosterone was instead converted into estradiol, and that changes in estrogen activity affected sleep architecture.

There is robust evidence that sleep-regulating areas of the brain are sensitive to sex hormones [30]. Estradiol has been shown to affect the ventrolateral preoptic nucleus (VLPO): this area showed decreased firing rates after estradiol therapy in mice, although changes in sex hormones in male rats had no effect [31, 32] and administration of estradiol into the VLPO resulted in increased physical activity in mice [33]. Furthermore, estradiol could affect the expression of orexin receptors, a sleep-stabilizing steroid. Orexin receptor expression changes with the estrous cycle in female rats as well as after removal and add-back of estradiol in female rats [34], as well as after gonadectomy or add-back of androgens in male rats [35]. Estradiol could also affect the serotoninergic activity in the dorsal raphe nucleus: exposure to estradiol and progestins has been found to alter expression of serotonin receptors [36]. Changes in firing rates in the VLPO and serotonin system could also affect the duration of REM sleep [37, 38] and non-REM sleep [39]. Furthermore, sex hormones are known to affect the suprachiasmatic nucleus (SCN) [40] and the dynamics of diurnal cortisol via the hypothalamus-pituitary-adrenal axis [41]. For a full review of neural mechanisms between sex hormones and sleep, see [30].

One explanation for the sex discrepancy in our results, showing that transmasculine participants show altered sleep but transfeminine participants do not, could also be found in possible sex differences in sex hormone sensitivity in the brain. The organizational-activational model of sexual differentiation (as posed by [42]) states that sex hormones can permanently affect neural development (so-called organizational effect), and they can also have a direct effect on neural activity (so-called activational effect). Studies in rodents show that this model might also apply to sex hormone effects on sleep: manipulation of sex hormone exposure during development in rats also alters the sensitivity to sex hormone effects on sleep. Male rats that underwent neonatal castration, resulting in lack of exposure to androgens, were found to show larger sleep changes after treatment with estradiol and progesterone than male rats that underwent endogenous puberty and were therefore exposed to androgens [43]. Vice versa, female rats that were treated with masculinizing postnatal testosterone treatment showed no sleep changes after sex hormone manipulations, whereas the untreated females which were exposed to endogenous puberty and female sex hormone exposure showed significant sleep changes after sex hormone changes [4]. These findings indicate that exposure to female sex hormones or lack of exposure to male sex hormones could possibly alter the brain’s sensitivity to sex hormone changes.

Interestingly, the demographic characteristics show that sleep quality improved and insomnia symptoms reduced after 3 months of GAHT in the TM group, although the found effect sizes are small to medium (ISI: 0.37 and PSQI: 0.39). This is in line with previous work from our group in a different cohort, where we found that insomnia and sleep quality both improve after 3 months of GAHT use, but show no lasting improvements after 12 months of GAHT use [44]. Within the current study, we seem to reproduce the existing paradox in the general population, where cisgender men show poorer sleep quality when measured using objective measures, but they report better sleep in subjective measures. Although there are no definitive explanations for this paradox, it is sometimes assumed that subjective and objective sleep are affected by different factors. For example, perceived sleep quality and insomnia symptoms could be more affected by anxiety and depression, which are more prevalent in women [45], whereas objective sleep could be more strongly affected by physiological and biological factors, such as sleep apnea.

The current study has a number of strengths. First of all, it was the first prospective study to investigate the effects of both feminizing and masculinizing GAHT on sleep architecture. In our setup, we used a single-electrode EEG device that could be used for ambulatory measurements, which means that participants were able to measure their sleep for multiple days at home. This strengthens the external validity of our sleep measurements, since participants slept in their normal home situations. Furthermore, our study design allows us to examine the effects of longer-term hormone use compared to previous work. Many studies assessing effects of exogenous sex hormones on sleep architecture in healthy participants focus on the effects of acute- or very short-term hormone exposure [8, 9], which means results typically display the acute effects, but not the longer-term effects of sex hormone use. Compared to these studies, 3 months of GAHT exposure shows the effects of longer-term hormone use. However, many transgender GAHT users will typically use GAHT for the rest of their lives [14], and future work should focus on longer-term effects of GAHT. The current study did not include outcomes after 12 months of GAHT use, since data collection was still ongoing, but future research from the current cohort will also provide more insight into the 12-month trajectory of sleep architecture changes after starting GAHT use.

There are also a number of limitations of this study. Firstly, it is possible that there is a form of selection bias in the participant group. Some participants reported that they found the sleep EEG device uncomfortable or that they forgot to charge or wear the device, and opted out of the measurements. In the supplementary materials, we have supplied an overview of the participant demographics (e.g. age, psychotropic medication use, and symptoms of depression, stress, or insomnia) of participants who participated in the objective sleep measures compared to those who did not (see Supplementary Table S3). These data show no clinically significant differences in symptoms of insomnia, depression, or perceived stress between the groups, indicating that the participants who opted in or out of the sleep EEG measurements were comparable. However, it is still possible that the participants with poor sleep were less likely to participate in the objective sleep measures or less likely to participate in the follow-up measurements altogether.

Secondly, we opted not to conduct full-head sleep polysomnography (PSG) measurements, which means we could not study REM sleep according to the “golden standard” measure, which includes, amongst other additional channels, an eye movement electrode, and we did not have the raw EEG data to measure sleep EEG microarchitecture, such as micro-arousals, sleep spindles, and eye movement. Furthermore, for EEG processing we relied on the automated sleep staging of the EEG device, which has an embedded deep neural network-based sleep stager. In a validation study, a (non-embedded) version of the sleep stager was compared to manual scoring of the EEG signal in [25]. This resulted in a Kappa score of 0.73, which is close to the human inter-rater agreement of about 0.75 [46]. The devices used in this study included EOG electrodes, but these were only used for the manual scoring and not used by the automatic sleep stager, allowing the neural network in the stager to be trained to detect REM sleep from single-channel EEG input only. When compared to full PSG manual scoring, the automatic stager showed even better agreement (κ = 0.76). As shown in [25], the sensitivity for REM sleep was 0.81, meaning the automated stager performs adequately in assessment of REM sleep. The current device did not equip EOG electrodes to assess REM sleep, relying on the automatic sleep stager to classify REM sleep. The performance of the automatic sleep stager in the device was compared to manual scoring of the devices’ raw EEG data in [47]. This study showed kappa scores of 0.65 to 0.67, compared to an inter-rater agreement of 0.69, indicating that the device’s automated stager performed equally to manual scoring. Our current methodology also did not equip oximetry measurements, meaning that possible changes in sleep apnea would not have been detected by the sleep EEG device, and we did not use any sleep apnea screening questionnaires. There have been cases of transgender men developing sleep apnea after starting testosterone [48, 49], and previous work has linked higher testosterone levels to increased risk of sleep apnea and reduced slow wave amplitude [50]. It is possible that the sleep architecture of the TM group after three months of GAHT was affected by sleep apnea, but we could not account for this possibility in the current study. For further study of sex hormones on sleep, we would also recommend further assessing sleep architecture changes using a multi-channel PSG measurement setup including oximetry.

Furthermore, as displayed in Table 1, numerous participants were using psychotropic medication, most commonly antidepressants or stimulants. There are known effects of psychotropic medication on sleep architecture [51], but after adjusting for psychotropic medication use, the results remained unchanged in terms of their direction and magnitude. Furthermore, none of the participants reported changing their psychotropic medication between the baseline measurement and 3-month follow-up. We therefore believe that the impact of psychotropic medication on our findings was small to minimal.

Altogether, our results show that sex hormones seem to change sleep architecture, but that the effects of sex hormones are dependent on one’s sex assigned at birth. Although these findings are from a relatively small population, they do provide evidence indicating there is a sex assigned at birth-specific predisposition for sensitivity to the effects of sex hormone fluctuations on sleep. This finding is important for research on transgender people, but it is also of major importance for studies on puberty, pregnancy, and menopause. Sleep is an important component of well-being, and decreased sleep quality is associated with poorer physical and mental health [52]. If sex hormone exposure, during development or during adulthood, could predispose one to be more sensitive to poor sleep during sex hormone transitions, this is of interest to public health.

Future research should focus on studying underlying mechanisms in sleep and sex hormones, mainly on the effects of estradiol and testosterone in sleep-promoting regions of the human brain, as well as on longer-term effects of GAHT on sleep architecture. Furthermore, future research should assess whether the worsening in sleep architecture in transmasculine persons has effects on their well-being, most importantly on the risk of depressive disorders.

## Supplementary Material

zsad249_suppl_Supplementary_Tables_S1-S3

## Data Availability

Data available on request.

## Preprint Repositories

This manuscript has been submitted as a preprint to MedRxiv under registration number MEDRXIV/2023/291701.
