# Supplementary material for: Influence of sex hormone use on sleep architecture in a transgender cohort
Source: Sleep. 2023 Sep 16;46(11):zsad249. doi: 10.1093/sleep/zsad249 (PMC10636253; doi:10.1093/sleep/zsad249)
Supplement: zsad249_suppl_Supplementary_Tables_S1-S3 [file zsad249_suppl_supplementary_tables_s1-s3.docx]

# Supplementary materials to accompany manuscript: “Influence of sex hormone use on sleep architecture in a transgender cohort: findings from the prospective RESTED study”

Margot W. L. Morssinkhof^1.2.3.4.^*, Ysbrand D. van der Werf^5.6.^, Odile A. van den Heuvel^1.5.6.^, Daan A. van den Ende^7.^, Karin van der Tuuk^8.^, Martin den Heijer^2.4.^, Birit F. P. Broekman ^1.3.9.^

1. Department of Psychiatry, Amsterdam UMC, location Vrije Universiteit Amsterdam, the Netherlands

2. Department of Endocrinology and Metabolism, Amsterdam UMC, location Vrije Universiteit Amsterdam, the Netherlands

3. Department of Psychiatry and Medical Psychology, OLVG, Amsterdam, the Netherlands

4. Center of Expertise on Gender Dysphoria, Amsterdam UMC, Vrije Universiteit Amsterdam, The Netherlands

5. Department of Anatomy and Neurosciences, Amsterdam UMC, Vrije Universiteit Amsterdam, Amsterdam, The Netherlands

6. Amsterdam Neuroscience, Compulsivity Impulsivity and Attention, Amsterdam, The Netherlands

7. Philips, Eindhoven, The Netherlands

8. Department of Obstetrics and Gynecology, University Medical Centre Groningen, Groningen, the Netherlands

9. Amsterdam Public Health, Mental Health Program, Amsterdam, the Netherlands

*Corresponding author: M. Morssinkhof, De Boelelaan 1117, 1081HV, Amsterdam. Email: [m.morssinkhof@amsterdamumc.nl](mailto:m.morssinkhof@amsterdamumc.nl)

## 1. Hormone form and dosage

The table below displays the form and dosage of GAHT used by the study participants, at the start of GAHT (e.g. after the baseline measurement) and at the 3-month follow up.

| Table S1. Hormone formulation prescribed at the start of GAHT and 3 month follow-up | | | | | |
| --- | --- | --- | --- | --- | --- |
|  | | **Transmasculine participants (n = 38 )** | | **Transfeminine participants  (n = 35)** | |
| Measurement | | Start of GAHT  n = 36 | At 3 month follow-up  n = 26 | Start of GAHT  n = 32 | At 3 month follow-up  n = 24 |
| Cycle regulation use (n, %) | Progestin only – oral | 4 (11%) | 3 (12%) | - | - |
|  | Progestin only – non-oral methods ^1.^ | 8 (22%) | 3 (12%) | - | - |
|  | Estradiol and progestin – combined oral contraceptives | 6 (17%) | 0 (0%) | - | - |
|  | None | 18 (50%) | 6 (23%) | - | - |
| Testosterone form (n, %) | Transdermal | 32 (89%) | 19 (73%) | - | - |
|  | Intramuscular – short-acting (esters) | 4 (11%) | 2 (8%) | - | - |
|  | Intramuscular – long-acting (undecanoate) | 0 (0%) | 5 (20%) | - | - |
| Estrogen form (n, %) | Oral | - | - | 17 (53%) | 14 (46%) |
|  | Transdermal – gel | - | - | 3 (9%) | 3 (13%) |
|  | Transdermal – spray | - | - | 0 (0%) | 0 (0%) |
|  | Transdermal – patches | - | - | 12 (38%) | 7 (29%) |
| Anti-androgen form (n, %) | GnRH analogues – short-acting | - | - | 25 (78%) | 13 (54%) |
|  | GnRH analogues – long-acting | - | - | 4 (13%) | 8 (33%) |
|  | Cyproterone actate | - | - | 3 (9%) | 3 (13%) |
| ^1.^ Methods include hormonal intrauterine devices, intramuscular injections, progestin implants | | | | | |

## 2. Smartsleep mode

The table below displays the outcomes stratified by sleep device setting and the estimated differences between the inactive and the active mode. Analyses show no significant differences between the active and inactive mode of the device.

| Table S2: sleep architecture during GAHT – with main predictor for devices in active mode. | | | | | | |
| --- | --- | --- | --- | --- | --- | --- |
|  | **TM** | | | **TF** | | |
|  | Means and SD or median and IQR | | Estimated difference active vs. inactive mode ^a.^ | Means and SD or median and IQR | | Estimated difference active vs. inactive mode ^a.^ |
| Setting | *Inactive* | *Active* |  | *Inactive* | *Active* |  |
| Measurement weeks | Baseline: n = 33  3MO: n = 15 | Baseline: n = 3  3MO: n = 11 | Beta, 95% confidence interval, p-value | Baseline: n = 31  3MO: n = 23 | Baseline: n = 1  3MO: n = 1 | Beta, 95% confidence interval, p-value |
| SOL (minutes) | 17.8 (7.96 to 33.1) | 20.02 (7.96 to 36.7) | -15.5% (-39.1% to 17.7%)  P = 0.31 | 13.98 (7.29 to 27.25) | 16.87 (12.22 to 26.85) | 3.21% (-10.95 to 19.58)  P = 0.68 |
| TST (hours) | 7.3 (1.4) | 7.6 (1.6) | 0.144 (-0.30 to 0.59)  P = 0.53 | 6.49 (1.33) | 6.64 (0.67) | 0.6594 (-0.251 to 1.559)  P = 0.15 |
| WASO (minutes) | 20.27 (12.8 to 38.4) | 21.0 (15.4 to 28.1) | 4.2% ( -18.7 to 33.4) p = 0.75 | 15.79 ( 9.83 to 37.54) | 21.66 (11.18 to 32.1) | -9.998% ( -47.56 to 54.47)  P = 0.70 |
| NRI | 1 (0 to 2) | 1 (0 to 2) | 6.96% ( -10.4% to 27.8%)  P = 0.46 | 1 (0 to 1.5) | 1 (0 to 2) | -17.26% (-44.38 to 23.26)  P = 0.35 |
| NRA | 27 (21 to 40) | 26.5 (19 to 43) | -3.1% ( -17.6 to 13.9)  P = 0.70 | 27 (41 to 18) | 40 (15.5 to 53.5) | - 13.80% (-41.08 to 26.17)  P = 0.45 |
| SWS (minutes) | 87 (31) | 93 (32) | -1.5 (-5.5 to 8.59)  P = 0.67 | 86 (30) | 82 (23) | -6.31 (-21.53 to 8.96)  P = 0.42 |
| % SWS | 20.0% (7.3%) | 21.0% (8.0%) | -0.04% ( -1.88 to 1.80)  P = 0.95 | 22.6 (8.05) | 20.82 (7.02) | -4.3247% (-9.2956 to 0.692)  P = 0.091 |
| REM sleep duration (minutes) | 118.9 (45.5) | 119.1 (38.9) | -9.90 (-23.27 to 3.49)  P = 0.149 | 114.26 (42.9) | 106.91 (19.67) | 7.938 (-20.63 to 36.28)  P = 0.58 |
| REM sleep latency (minutes) | 82 (33.5 to 125.5) | 68 (28.8 to 101.5) | -1.9% (-29.6 to 39.3)  P = 0.95 | 81.00 (22.5 to 111.5) | 89.0 (39.5 to 95.0) | 29.74% (- -42.66 to 90.51)  P = 0.53 |
| ^a.^ Analyzed using a linear mixed model with measurement phase and device setting as fixed predictors and a random intercept per participant, with inactive mode as the reference category. | | | | | | |

## 3. Participation and non-participation in objective sleep measurements

The table below displays demographic descriptions and questionnaire outcomes of participants who contributed sleep device measurements to the final dataset and participants who did not. It is possible that participants took part in the objective sleep measurements, but that the measurements were of low quality and that they were excluded, which means the participant’s measurements are therefore not present in the final dataset.

| Table S3. Demographic and clinical characteristics of participants opting in and out of sleep device measurements. IQR = Interquartile range. | | | | | |
| --- | --- | --- | --- | --- | --- |
|  |  | Not present in objective sleep measurements | Present in baseline objective sleep measurements only | Present in 3MO objective sleep measurements only | Present in baseline and 3MO |
| n |  | 26 | 23 | 5 | 45 |
| Age | *Median, IQR* | 22 (20 to 24.5) | 24 (21.5 to 26) | 28 (19 to 30) | 24 (22 to 27) |
| Group (n, %) | *TM* | 13 (50%) | 12 (52%) | 2 (40%) | 24 (53%) |
|  | *TF* | 13 (50%) | 11 (48%) | 3 (50%) | 21 (47%) |
| Psychotropic medication use (n, %) | *Yes* | 5 (19%) | 2 (9%) | 1 (20%) | 9 (20%) |
|  | *No* | 21 (81%) | 21 (91%) | 4 (80%) | 36 (80%) |
| *Scores at baseline* | | | | | |
| ISI scores  (range 0 to 28) | *Median IQR missings (n)* | 7.5 4.75 to 10.25 2 | 5.5 3.25 to 10.5 1 | 6.0 4.0 to 6.0 0 | 6.0 3.0 to 9.0 0 |
| PSQI scores  (range 21) | *Median IQR missings (n)* | 7  4.25 to 9.0 4 | 5 4 to 9 4 | 4 4 to 6 0 | 6 4 to 8 4 |
| IDS-SR scores  (range 0 to 84) | *Median IQR missings (n)* | 15.5 10 to 26 4 | 12 8 to 25.75 1 | 9 8 to 25 0 | 12 8 to 21 0 |
| PSS scores  (range 0 to 40) | *Median IQR missings (n)* | 13 9.5 to 16.25 2 | 12 8.25 to 17 1 | 11 6 to 13 0 | 10 8 to 16 0 |
